# Multimeric Assembly of Host-Pathogen Adhesion Complexes Involved in Apicomplexan Invasion

**DOI:** 10.1371/journal.ppat.1004120

**Published:** 2014-06-12

**Authors:** May M. Paing, Niraj H. Tolia

**Affiliations:** Department of Molecular Microbiology and Microbial Pathogenesis, Washington University School of Medicine, Saint Louis, Missouri, United States of America; University of Wisconsin Medical School, United States of America

Apicomplexan parasites are the causative agents of diseases that include malaria, toxoplasmosis, and coccidiosis. These obligate intracellular parasites have evolved to use a conserved mechanism for host-cell invasion. The apicomplexan phylum is defined by the presence of micronemes and rhoptries, which are distinct organelles located at the apical end of the parasite. These organelles secrete molecules necessary for host-cell invasion [1]. Apicomplexan parasites can invade disparate cell types, including hepatocytes, erythrocytes, lymphocytes, macrophages, and cells lining the digestive tract. Unlike viruses and intracellular bacteria, apicomplexans actively invade host cells without relying on host uptake pathways. As such, host-cell sensing and subsequent invasion are driven entirely by the parasite in a dynamic and rapid process. Intracellular residence protects the parasite from immune attack and enables parasite replication prior to host-cell lysis and subsequent invasion of neighboring host cells.

The repertoire of ligand-receptor complexes utilized by parasites for entry into host cells is diverse. Some interactions occur through cell-specific receptors resulting in high-affinity interactions, while others occur through multiple lower-affinity interactions via surface moieties found on several cell types. Receptor-specific and general cell binding may explain host-cell tropism of different pathogens, although additional factors are important. There is growing evidence that multimeric assembly of parasite ligands and host surface molecules strengthens the host-pathogen interactions necessary for invasion. We discuss recent work that has advanced our knowledge of the assembly of adhesive complexes from two critical apicomplexan pathogens and highlight areas of research that require further investigation.

## Concepts That Define Multimeric Assembly of Complexes

Affinity, avidity, and valency are necessary concepts to define receptor-ligand interactions. The strength of attachment for two binding partners is determined by the affinity of individual binding sites and the number of interacting binding sites (valency). Avidity is the accumulated strength of multiple affinities from multivalent binding sites. The avidity of a multivalent complex is typically far greater than the sum of the individual affinities because of synergism between independent sites: dissociation at one site will be compensated by a bound second site, leading to rapid reassociation at the first site. Parasite ligands have evolved to increase both affinity and valency, resulting in high avidity that is necessary to create strong interactions that anchor parasites to host cells. Further adhesion strengthening is achieved through increased local surface concentration of ligands resulting in multiple focused interactions. In this review, we highlight parasite protein ligands that have evolved diverse methods to form high-avidity complexes for invasion. Specific mechanisms include utilizing repeat units, tandem duplication of adhesive domains, and homo- or hetero-oligomerizing with multimeric host receptors upon engagement.

## *Plasmodium* Sporozoite Motility and Invasion

*Plasmodium falciparum* sporozoites invade the cells of the mosquito salivary glands prior to injection into the human host. Once injected, sporozoites migrate through the dermis, enter capillaries, traverse Kupffer cells that form the endothelial lining of the liver, and finally invade hepatocytes. The best-characterized invasion complexes with roles during these processes are mediated by thrombospondin-related anonymous protein (TRAP) and circumsporozoite protein (CSP).

**Figure 1:**
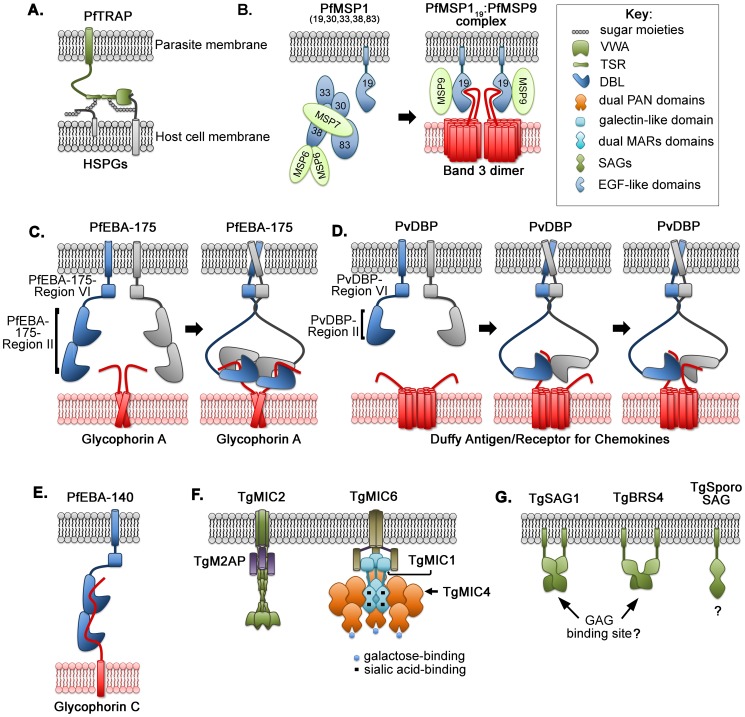
Multimeric assembly, clustered interactions, and molecular complexes between parasite ligands and host-cell receptors for invasion. (**A**) PfTRAP engagement with heparan sulphate proteoglycans (HSPGs) on the hepatocyte surface; (**B**) proteolytic processing and shedding of PfMSP1 exposes the 19 kDa fragment (MSP1_19_) that forms an invasion complex with MSP9 and the band 3 homodimer; (**C**) assembly of two PfEBA-175 monomers around dimeric glycophorin A of erythrocytes; (**D**) stepwise multimeric assembly of two PvDBP with two Duffy antigen/receptor for chemokines on reticulocyte surface; (**E**) monomeric interaction between PfEBA-140 and glycophorin C on erythrocytes; (**F**) proposed complexes of TgMIC2 and TgM2AP and of TgMIC1, TgMIC4, and TgMIC6 on the parasite surface; (**G**) variations in oligomeric states of GPI-anchored surface antigens (SAGs) create distinct interaction sites.

*P. falciparum* TRAP (PfTRAP) has a role in sporozoite gliding motility, salivary gland invasion, and sporozoite infectivity [2]. This adhesin is stored within micronemes and is released onto the cell surface at the anterior tip upon contact with a host cell. PfTRAP contains two adhesive domains: a von Willebrand factor type A (VWA) domain and a thrombospondin type-I repeat (TSR) domain. Attachment to host cells occurs through both the VWA domain, which is similar to the I-domains of integrins that are important for magnesium cation coordination, and the TSR domain that binds to abundantly expressed heparan sulphate proteoglycans (HSPGs) on the hepatocyte surface [3], [4]. Individually, each domain or repeat binds to its respective interacting molecule, and the overall avidity of binding is likely increased by the tandem clustering of multiple repeats and domains (Figure 1A).

*P. falciparum* CSP (PfCSP) is the most abundant antigen expressed on the surface of sporozoites and is the major antigen of a pre-erythrocytic malaria vaccine that confers limited protection [5]. PfCSP is anchored to the surface via a glycosylphosphatidylinositol (GPI) moiety and is crucial for sporozoite infection of hepatocytes [6]. PfCSP shares with PfTRAP the presence of TSR repeats [7]. The seven degenerate sulphatide binding motifs in the PfCSP TSR repeats bind the abundantly expressed HSPGs on host cells, resulting in high-avidity binding driven by the tandem duplication of individual repeats.

## *Plasmodium* Merozoite Invasion of Red Blood Cells

The *P. falciparum* erythrocytic cycle begins with merozoite recognition and invasion of red blood cells (RBCs). Initial binding to the RBC is mediated by merozoite surface proteins (MSPs). The most abundant of the merozoite surface proteins is the complex of GPI-anchored MSP1 noncovalently attached to MSP6 and MSP7 [8]. MSP1 is proteolytically processed upon merozoite egress from a previously infected host cell. The multipartite MSP1 complex resides on the surface of the free merozoite and is shed at the time of RBC invasion to expose the C-terminal GPI-anchored MSP1_19_ in complex with MSP9 for RBC entry. The MSP1_19_/MSP9 multimer likely stabilizes and enhances the avidity of binding to the most abundant RBC membrane protein, the band 3 homodimer [9]. Engagement of band 3 is thought to be mediated by two epidermal growth factor (EGF)-like domains in MSP1_19_ (Figure 1B).

The erythrocyte binding like (EBL) family has a defined role in recognition of and attachment to erythrocytes by engaging specific erythrocyte receptors [10]–[12]. EBL ligands are released from micronemes onto the apical surface of merozoites during invasion [13]. These proteins contain one or two conserved Duffy binding like (DBL) receptor-binding domains (Region II), a cysteine-rich domain (Region VI), and a transmembrane domain [14]. The EBL ligands in *P. falciparum* contain two DBL domains in Region II and include PfEBA-175, PfEBA-140/BAEBL, PfEBL-1, and PfEBA-181/JESEBL. Structural and biophysical studies have elucidated mechanisms of receptor engagement for members of this family.

The first member of the family to be structurally characterized was PfEBA-175 (Figure 1C). Two PfEBA-175 monomers dimerize around the glycosylated extracellular domains of glycophorin A dimers [15], [16], resulting in a high-avidity interaction [17], [18]. The sialylated glycans of glycophorin A are recognized by sialic acid-binding pockets created at the interface between Region II of each monomer [16]. The complex assembly requires both DBL domains of each monomer and is enhanced by additional regions of PfEBA-175 [17], [18].

In *P. vivax*, the Duffy-binding protein (PvDBP) contains a single DBL domain that binds to the Duffy antigen/receptor for chemokines (DARC) (Figure 1D), a nonsignaling G-protein-coupled receptor on reticulocytes [19]–[21]. Even though the DBL domain architectures of PvDBP and PfEBA-175 are different, these ligands have a similar mechanism of receptor engagement. PvDBP is monomeric in the absence of DARC, and DARC binding drives dimerization of PvDBP [22]. Examination of multimeric assembly in solution and capture of PvDBP∶DARC complexes by crystallography revealed the formation of a heterotrimer of two PvDBPs bound to one DARC, followed by a heterotetramer of two PvDBPs engaging two DARCs [23]. These complexes suggest stepwise assembly, which is likely to be cooperative, leading to a high-avidity PvDBP∶DARC interaction.

The two DBL domains of PfEBA-140 Region II independently bind to sialylated glycans of glycophorin C on erythrocytes [24]–[26]. While PfEBA-175 and PvDBP dimerize upon receptor engagement, PfEBA-140 may contact glycophorin C as a monomer (Figure 1E) [25], [26]. Additional studies are necessary to examine if multimeric assembly occurs upon receptor binding or if oligomerization is an important determinant of receptor specificity. PfEBA-140 Region II has also evolved novel glycan-binding pockets, distinct from those in PfEBA-175, and these do not require dimerization [25], [26].

Disruption of multimeric assembly is an effective method for antibody neutralization of parasite growth. An antibody that binds to the PfEBA-175 dimer interface and receptor-binding sites effectively disrupts binding to glycophorin A and blocks *P. falciparum* invasion [27]–[29]. Similarly, the residues at the dimer interface and DARC-binding groove are targeted by naturally acquired antibodies correlated with disruption of PvDBP binding [22], [23], [30]. These studies suggest that assembly of ligands around receptors leading to high-avidity interactions is an important determinant of receptor binding and that immune targeting of oligomeric interfaces in addition to receptor-binding pockets leads to protection.

## Multimeric Micronemal Protein Complexes of *Toxoplasma gondii*

The microneme proteins (MICs) in *Toxoplasma gondii* preassemble in the endoplasmic reticulum and form complexes prior to transiting to the micronemes. The propensity to form oligomers with different combinations of partners likely allows the parasite to expand the receptor repertoire or fine-tune the specificity of receptor binding. To date, three major complexes have been identified and functionally characterized in *T. gondii* attachment to host cells. First, microneme protein 2 (TgMIC2), a member of the conserved TRAP family, is found in a heterohexameric complex with MIC2-associated protein (TgM2AP) (Figure 1F) and plays a fundamental role in gliding motility and host-cell attachment [31], [32]. Each TgMIC2 monomer binds one TgM2AP monomer via the TSR repeats in TgMIC2 [33]. Second, TgMIC8, which complexes with the lectin-like TgMIC3, is essential for rhoptry secretion and invasion [34]. Third, TgMIC6 forms a multimeric complex with two adhesins, TgMIC1 and TgMIC4, and contributes to invasion in vitro and virulence in vivo [35]–[37].

The TgMIC1∶4∶6 complex has been the most characterized structurally (Figure 1F). Although TgMIC1 was classified as a TRAP family member, structural studies of the N-terminal repeat units and C-terminal domain have revealed novel adhesion modules [36], [38]. The C-terminal galectin-like domain of TgMIC1 stabilizes the interaction with the EGF domains of TgMIC6, which in turn anchors the complex via a transmembrane domain [37], [38]. The N-terminus of TgMIC1 contains two micronemal adhesive repeats (MAR) that bind sialic acid [39]. TgMIC1 forms a disulfide-linked trimer, and each TgMIC1 monomer further engages a TgMIC4 monomer, creating a heterohexamer. The two tandem apple domains of TgMIC4 bind galactose-containing glycans [39]. The duplication of MAR repeats and apple domains, coupled with heterohexamerization, likely results in high avidity by increased valency for sialic acid and galactose.

## *Toxoplasma* Surface Antigens

Surface antigen glycoproteins (SAGs) and SAG-related sequence proteins (SRS) are abundant and widely distributed GPI-anchored adhesins on the *T. gondii* surface at multiple stages of the life cycle [40]–[43]. They are optimally positioned for low-affinity, lateral interactions with the host-surface glycosaminoglycans, which act as receptors for *Toxoplasma* invasion [44], [45]. Crystal structures of SAGs revealed varying levels of dimerization: SAG1 forms a parallel homodimer with an extensive dimer interface [46], Bradyzoite-specific surface antigen (BRS4) exhibits a smaller dimer interface [47], while the SAG expressed in sporozoite stage (SporoSAG) is monomeric (Figure 1G) [48]. Variation in oligomeric state may impact receptor binding as the SAG1 and BRS4 dimers create basic pockets implicated in glycosaminoglycan engagement. The basic pocket is replaced by an acidic cap in SporoSAG, and the receptor moiety engaged is unclear. It is plausible that, like EBL-ligands, receptor binding induces or stabilizes dimerization of SAGs, although further structural studies in solution are necessary.

In summary, the organization of parasite ligands at the site of invasion is promoted by multivalent, high-avidity interactions with host-cell receptors and surface moieties. The strength of attachment can be further increased by clustering of adhesive complexes. This combination of clustered interactions and multimeric complexes not only ensures the parasite's successful entry into the host cell but also likely promotes evasion from the host's immune response by burying potentially protective antigenic epitopes. Increased avidity has been demonstrated for some but not all multivalent complexes, and future studies are necessary to clearly identify the effect of multimeric assembly on binding and avidity in cases in which this information is lacking. Assembly can also activate or enhance downstream signaling processes in other systems, and further studies are needed to decipher whether signaling is triggered by multimeric assembly during invasion. The structural determination of critical interfaces in ligand-receptor binding and the biochemical and biophysical elucidation of multimeric assembly mechanisms will provide novel perspectives on how the invasion process is manifested and regulated. This information will identify novel ways to block pathogen entry into host cells.
